# Cobalt-Catalyzed (Hetero)arylation of Saturated Cyclic Amines with Grignard Reagents

**DOI:** 10.3390/molecules23061449

**Published:** 2018-06-14

**Authors:** Baptiste Barré, Laurine Gonnard, Amandine Guérinot, Janine Cossy

**Affiliations:** Laboratoire de Chimie Organique, Institute of Chemistry, Biology and Innovation (CBI)-UMR 8231 ESPCI Paris, CNRS, PSL Research University 10, rue Vauquelin 75231 Paris CEDEX 05, France; baptiste.barre@outlook.com (B.B.); laurine.gonnard@gmail.com (L.G.)

**Keywords:** *N*-heterocycle, piperidine, pyrrolidine, azetidine, cross-coupling, cobalt, Grignard reagent

## Abstract

(Hetero)aryl substituted saturated cyclic amines are ubiquitous scaffolds in biologically active molecules. Metal-catalyzed cross-couplings between halogeno *N*-heterocycles and organometallic species are efficient and modular reactions to access these attractive scaffolds. An overview of our work concerning the cobalt-catalyzed arylation of iodo-substituted cyclic amines is presented.

## 1. Introduction

**Figure 1:**
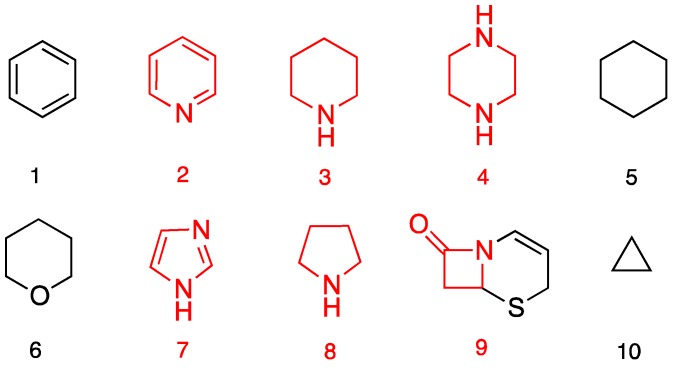
Top 10 of the most frequent rings in drugs.

The analysis of druglike chemical space is of outstanding importance in medicinal chemistry. In a recent report, Taylor et al. studied the occurrence of ring frameworks in approved drugs listed in the FDA orange book and established a top 100 of the most frequent ring systems present in small molecule drugs [1]. Although the benzene ring holds the first place in the classification, *N*-heterocycles are ubiquitous. Among them, pyridine is ranked second, piperidine third, piperazine fourth, imidazole seventh and pyrrolidine eighth. Worthy of note, while azetidine itself is not present in the classification, a bicylic ring system incorporating a β-lactam moiety can be found at position nine (Figure 1).

**Scheme 1:**
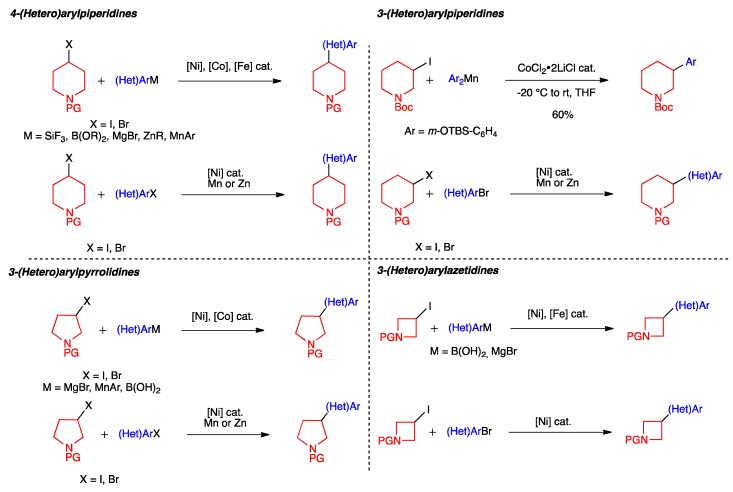
Metal-catalyzed cross-coupling reactions between halogeno *N*-heterocycles and (hetero)aromatic organometallic reagents.

Among the saturated *N*-heterocycles, 4-arylpiperidines, 3-arylpiperidines, 3-arylpyrrolidines and 3-arylazetidines are attractive scaffolds in drug discovery, exhibiting a broad range of biological activities [2,3,4,5,6,7,8]. Due to these interesting properties, numerous methods have been developed to access these aryl substituted *N*-heterocycles, either through ring formation or arylation of cyclic compounds [9,10,11,12,13]. The latter approach could be considered more convergent when molecular diversity is targeted and, particularly, metal-catalyzed cross-couplings involving (pseudo)halogeno-*N*-heterocycles have emerged as a powerful strategy to generate libraries of arylated *N*-heterocycles. In this field, tremendous efforts have been made for the arylation of 4-(pseudo)halopiperidines and numerous metal-catalyzed cross-couplings with organometallic reagents have been reported. Thus, Hiyama [14,15] Suzuki [16,17,18,19,20], Kumada-Corriu [21,22,23,24] and Negishi [25,26,27,28,29,30,31,32,33] couplings have been used to synthesize 4-arylpiperidines using nickel, iron and cobalt catalysts [34]. In addition, nickel-catalyzed reductive coupling involving 4-halopiperidines and (hetero)aryl halides have also been described in the literature [35,36,37,38,39]. In contrast, examples of arylation of 3-halopiperidines are scarce. Knochel et al. recently disclosed a cobalt-catalyzed cross-coupling between 3-iodo-*N*-Boc-piperidine and diarylmanganese reagents to furnish the 3-aryl piperidine in 60% yield [34]. 3-Arylpiperidines could also be formed through nickel catalyzed reductive coupling using aryl bromides [38,39,40]. Similarly, various metal-catalyzed cross-coupling with aryl organometallics [34,41,42,43,44] and metal-catalyzed reductive coupling with aryl halides [38,45] have been used to access 3-arylpyrrolidines and the arylation of 3-haloazetidines has received increasing attention over the past few years. Most of the examples involve nickel-catalyzed Suzuki cross-coupling with aryl boronic acids [46] but 3-arylated azetidines could be obtained from 3-iodoazetidines using iron-catalyzed coupling with aryl Grignard reagents [23,47]. Reductive cross-couplings between 3-iodoazetidine and aryl bromides were also employed to access 3-aryl- azetidines [33,44,48,49,50] (Scheme 1). 

**Scheme 2:**
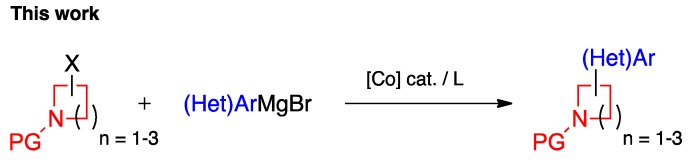
Cobalt-catalyzed cross-coupling reactions between halogeno *N*-heterocycles and (hetero)aromatic Grignard reagents.

Despite the variety of reactions existing for the arylation of saturated *N*-heterocycles from halogeno precursors, a unified method allowing the arylation of 4- and 3-halopiperidines, 3-halo-pyrrolidines and 3-haloazetidines was still needed. During the course of our studies toward the development of sustainable methods to access biologically relevant scaffolds, cobalt-catalyzed arylation of saturated halogeno *N*-heterocycles was investigated [51,52,53]. Herein, we report an overview of our work concerning the arylation of 4-iodopiperidines and 3-iodopiperidines, -pyrrolidines and -azetidines. The reactions are efficient, versatile, chemoselective and involve a non-expensive catalytic system (Scheme 2) [54,55]. 

## 2. Arylation of 4-Halopiperidines

The reactivity of 4-halopiperidines with aryl Grignard reagents was evaluated to access 4-aryl- piperidines. When *N*-Boc-4-iodo piperidine **1a** was treated with phenylmagnesium bromide in the absence of any metal catalyst, no conversion of the iodide was observed and the starting material was fully recovered (Table 1, entry 1). Pleasingly, when Co(acac)_3_ (5 mol %) was introduced in association with *N*,*N*-tetramethylethylenediamine (TMEDA) (6 mol %) as a ligand, the iodo piperidine **1a** was partially transformed to the coupling product **2a** (**1a**/**2a** = 79:21) (Table 1, entry 2). The replacement of TMEDA by (*R*,*R*)-tetramethylcyclohexanediamine (TMCD) increased the conversion of **1a** (**1a**/**2a** = 87:13) (Table 1, entry 3). Finally, changing Co(acac)_3_ for CoCl_2_ proved beneficial, allowing a full conversion of **1a** into the 4-phenylpiperidine, which could be isolated with a good 81% yield (Table 1, entry 4). 

With these optimized conditions in hand, the influence of the *N*-protecting group on the cross-coupling outcome was investigated. A *N*-tosyl group was tolerated but the corresponding arylated *N*-tosyl piperidine **2b** was isolated with an inferior yield compared to the *N*-Boc piperidine **2a** (69% *versus* 81%) and two equivalents of the Grignard reagent were required to ensure full conversion of the iodide (Table 1, entry 5). With a *N*-benzyl protected iodopiperidine, the coupling proved more difficult, probably due to the coordination of the nitrogen atom to the metal catalyst, and the use of 50 mol % of TMCD was needed to obtain a satisfactory yield in **2c** (66%) (Table 1, entry 6). The *N*-Boc 4-bromopiperidine **1b** was less reactive than its iodide counterpart and an increased amount of the Grignard reagent had to be added to reach full conversion (Table 1, entry 7). In light of these results, the best conditions to obtain 4-phenylpiperidine involve the use of *N*-Boc 4-iodopiperidine (1 equiv), CoCl_2_ (5 mol %), TMCD (6 mol %) and phenylmagnesium bromide (1.2 equiv) in THF. No syringe pump was needed, the dropwise addition of the Grignard reagent was performed at 0 °C and stirring was continued for 3 h at room temperature. With these conditions in hand, the scope of the arylation was investigated using *N*-Boc iodopiperidines.

**Scheme 3:**
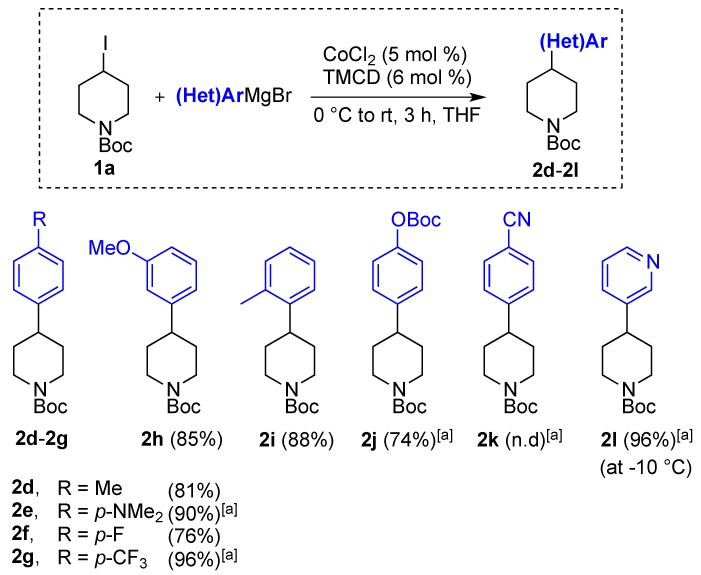
Scope of the cross-coupling involving *N*-Boc-4-iodopiperidine **1a** and various aryl Grignard reagents. ^[a]^ Grignard reagents prepared as ArMgBr.LiCl were used [56].

A range of aryl magnesium bromides was used in the cross-coupling with *N*-Boc 4-iodo- piperidine **1a**. The electronic properties of the substituents present on the phenyl ring had little influence on the reaction as both electron-donating (*p*-Me, *p*-NMe_2_) and electron-withdrawing (*m*-OMe, *p*-F, *p*-CF_3_) groups were well tolerated. In some cases, the Grignard reagent was prepared as an LiCl complex according to a method reported by Knochel et al. using a catalytic amount of DIBAL-H and a stoichiometric amount of LiCl [56]. These Grignard reagents exhibited similar reactivity to the classical ones under the coupling conditions. The coupling was not sensitive to steric hindrance and *o*-tolylmagnesium bromide reacted smoothly with iodopiperidine **1a** to deliver **2i** in a good 88% yield. The formation of the 4-arylpiperidine **2j** possessing a carbonate substituent on the phenyl ring highlighted the chemoselectivity of the cross-coupling. However, in the presence of a cyano group, the reaction turned sluggish leading to a poor conversion of **1a** (**1a**/**2k** = 70:30) that did not allow the isolation of the corresponding 4-arylpiperidine **2k**. A poisoning of the catalyst by coordination of the nitrile group could account for this limitation. Pleasingly, 3-pyridylmagnesium bromide was efficiently coupled to iodopiperidine **1a**, a decrease in temperature being the key to achieve a high yield in **2l** (96%). Despite its Lewis basicity, the nitrogen atom present on the pyridine moiety does not seem to interfere with the cobalt catalyst (Scheme 3). 

## 3. Arylation of 3-Halopiperidines

**Scheme 4:**
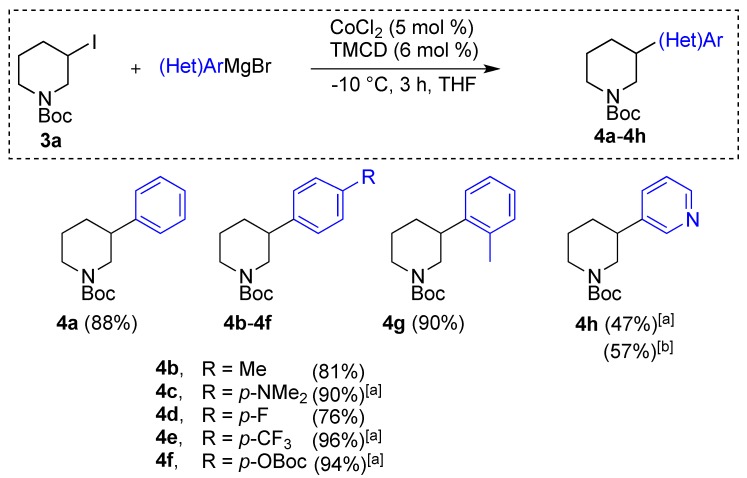
Scope of the cross-coupling involving *N*-Boc-3-iodopiperidine **3a** and various aryl Grignard reagents. ^[a]^ Grignard reagents prepared as ArMgBr.LiCl were used [56]. ^[b]^ Using 50 mol % of TMCD.

As previously mentioned, examples of cross-coupling involving 3-halopiperidines are scarce in the literature. When the previously developed conditions [CoCl_2_ (5 mol %), TMCD (6 mol %), 0 °C to rt] were used to perform the coupling between *N*-Boc 3-iodopiperidine **3a** and phenylmagnesium bromide, a full conversion of **3a** was observed, but the desired arylated compound was formed together with several non-identified side products. Lowering the temperature to -10 °C was crucial to allow a selective reaction to occur and, under these conditions, piperidine **4a** was isolated in a good yield (88%). A variety of aryl Grignard reagents differing from the nature of their substituents on the phenyl ring were used with iodopiperidine **3a**, delivering the corresponding 3-arylpiperidine with good to excellent yields (81–94%). The introduction of a pyridyl group at the C3 position was possible, but a moderate yield in the coupling product **4h** was obtained (47%). Increasing the amount of the TMCD ligand (50 mol %) only slightly improved the result (57%) (Scheme 4). 

**Scheme 5:**
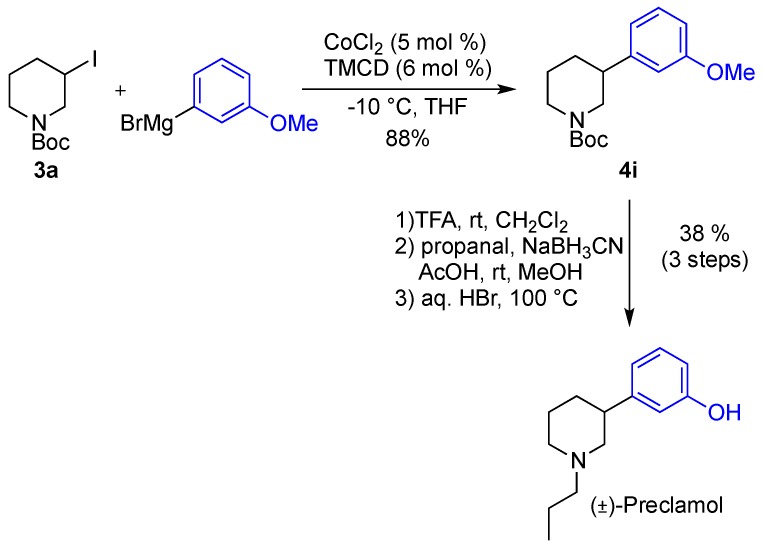
Synthesis of (±)-preclamol.

The cross-coupling was then used at the key step in a concise synthesis of the antipsychotic (±)-preclamol [57,58,59]. Treatment of the *N*-Boc 3-iodopiperidine **3a** with *m*-methoxyphenylmagnesium bromide in the presence of the CoCl_2_/TMCD catalytic system afforded the 3-arylated piperidine **4i** (88%). After the amine deprotection and reductive amination, the *N*-propylpiperidine was obtained. Finally, the cleavage of the methoxyether under acidic conditions (HBr) delivered (±)-preclamol (33% overall yield over four steps) (Scheme 5).

**Scheme 6:**
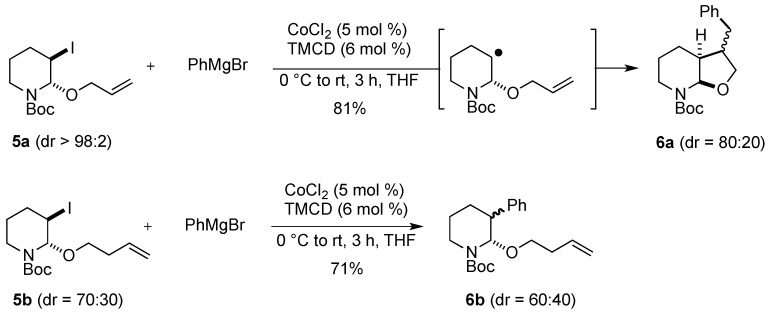
Cross-coupling reactions involving radical clocks **5a** and **5b.**

In the literature, the formation of radical species during cobalt-catalyzed cross-coupling has been observed on several occasions [60,61,62,63]. To determine if radical intermediates were present during the arylation of 3-iodopiperidines under our optimized conditions, two radical clocks were synthesized. When the disubstituted 3-iodopiperidine **5a** possessing an *O*-allyl group at C2 was treated with phenylmagnesium bromide in the presence of CoCl_2_ and TMCD, the bicyclic compound **6a** was formed exclusively (81%, dr = 80:20) (Scheme 6). This product results from a 5-*exo*-*trig* cyclization prior to the cross-coupling. On the contrary, iodopiperidine **5b**, possessing an additional carbon atom on the C2 pendant chain, was transformed in the 3-phenylpiperidine **6b** resulting from a direct cross-coupling at C3 (Scheme 6). These results could suggest that transient radical intermediates were formed during the coupling. The evolution of these species (cyclization then cross-coupling *versus* direct cross-coupling) would depend on the kinetic of the cyclization, the 5-*exo*-*trig* cyclization being approximately 1000 times faster than the 6-*exo*-*trig* cyclization.

## 4. Arylation of 3-Iodopyrrolidines

**Scheme 7:**
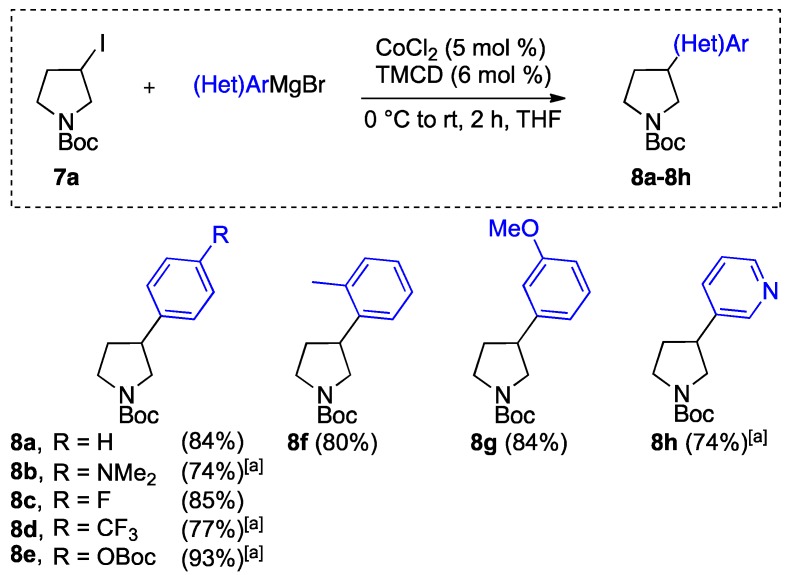
Cross-coupling of 3-iodopyrrolidine **7a** with various aryl Grignard reagents. ^[a]^ Grignard reagents prepared as ArMgBr.LiCl were used [56].

The cross-coupling was then successfully extended to the arylation of 3-iodopyrrolidines. A library of 3-arylpyrrolidines was prepared using a similar catalytic system to the one used for the arylation of iodopiperidines [CoCl_2_ (5 mol %), TMCD (6 mol %)]. Whatever the nature of the aryl Grignard reagent, the 3-arylpyrrolidines were obtained with good to excellent yield (74%–93%) (Scheme 7) [56]. 

**Scheme 8:**
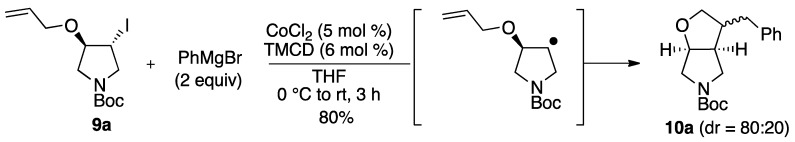
Cross-coupling involving radical clock **9a.**

When iodopyrrolidine **9a** bearing an *O*-allyl chain at C4 was involved in the cross-coupling, the bicyclic compound **10a** was obtained as the sole product with a good yield of 80% (dr = 80:20) (Scheme 8). The existence of this 5-*exo*-*trig* cyclization prior to the cross-coupling supports the hypothesis of radical intermediate formation during the reaction.

## 5. Arylation of 3-Iodoazetidines

**Scheme 9:**
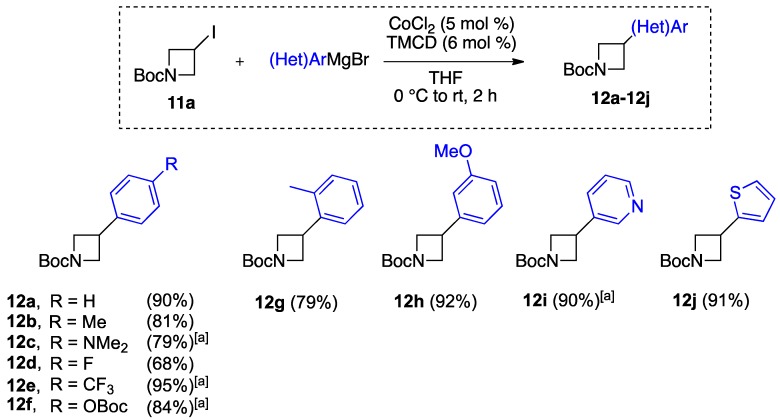
Cross-coupling of 3-iodoazetidine **11a** with various aryl Grignard reagents. ^[a]^ Grignard reagents prepared as ArMgBr.LiCl were used [56].

Pleasingly, the same catalytic system proved also efficient for the arylation of *N*-Boc 3-iodoazetidine **11a**. A broad range of aryl groups were efficiently introduced at the C3 position of the azetidine. Interestingly, heteroaryl Grignard reagents incorporating either a pyridine or a thiophene moiety were well tolerated, leading to the corresponding azetidines **12i** and **12j** with excellent yields of ca. 90% (Scheme 9) [56]. 

**Scheme 10:**
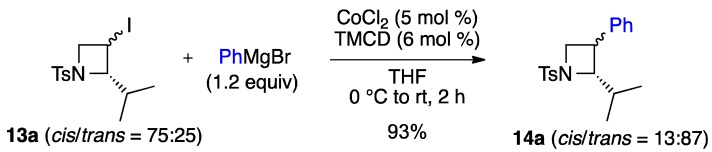
Diastereoselectivity of the cross-coupling reaction.

The reactivity of 2,3-disubstituted azetidines was then investigated and a mixture of *cis*- and *trans*-iodoazetidines **13a** (*cis*-**13a**/*trans*-**13a** = 75:25) was treated with phenylmagnesium bromide in the presence of CoCl_2_ and TMCD. The desired arylated product was isolated in 93% yield as a mixture of diastereomers, the *trans* diastereomer being the major product (*cis*-**14a**/*trans*-**14a** = 13:87) (Scheme 10). The inversion of the *cis*/*trans* ratio could be due to the formation of a radical intermediate at the C3 position that allowed a diastereoconvergent coupling to occur. However, an epimerization of the organocobalt species resulting from the oxidative addition step could not be excluded.

## 6. Mechanistic Hypothesis

**Scheme 11:**
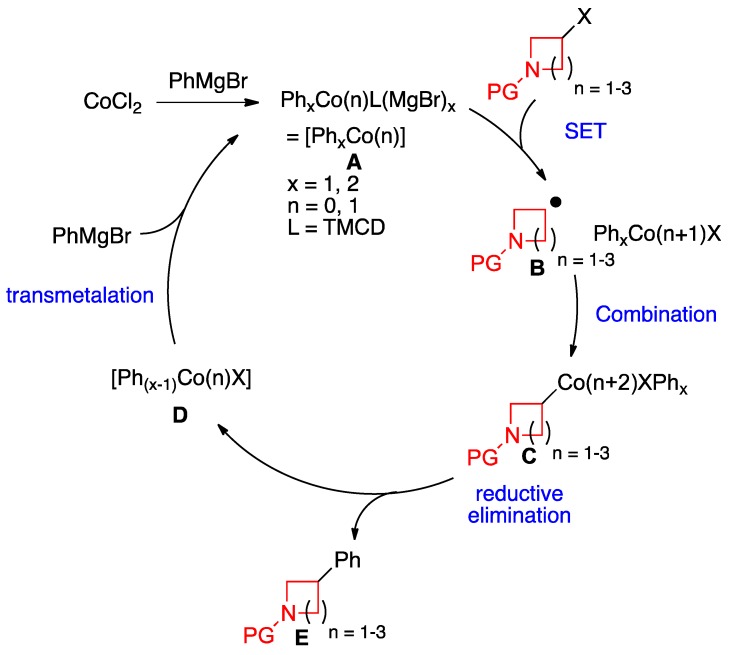
Hypothetical mechanism.

Based on literature reports [61] and on our observations, the mechanism depicted in Scheme 11 can be proposed. At first, a reduction of the Co(II) complex into the active species would be performed by the Grignard reagent to deliver the active catalyst **A** [Ph_x_Co(n) (*n* = 0, I)], which oxidation state remains uncertain. A Single Electron Transfer (SET) from this complex to the iodo *N*-heterocycle would deliver a Co(n+1) complex together with the radical intermediate **B**. A subsequent combination of the Co(n+1) complex with radical **B** would led to an organocobalt (n+2) intermediate that would give the product after reductive elimination. A transmetalation between complex **D** and PhMgBr would finally regenerate the active catalyst.

## 7. Conclusions

In conclusion, an efficient and simple cobalt-catalyzed arylation of saturated iodo-*N*-heterocycles with Grignard reagents was developed. A diversity of *N*-arylated heterocycles was thus prepared from the iodide precursors using a unique catalytic system composed of CoCl_2_ and TMCD. The process is non-expensive, versatile, chemoselective and diastereoselective. The reaction has been used as the key step in a short synthesis of (±)-preclamol. In the future, this cross-coupling could be of high synthetic value to access biologicaly active molecules embedding saturated *N*-heterocycles.

## Figures and Tables

**Table 1:**
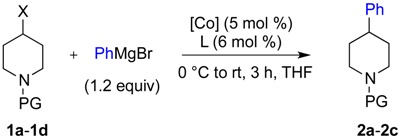
Cobalt-catalyzed cross-coupling between 4-halopiperidines and phenylmagnesium bromide.

Entry	1 (X, PG)	[Co]	L ^[a]^	1/2 ^[b]^	2 (yield) ^[c]^
1	**1a** (I, Boc)	-	-	**1a**/**2a** = 100:0	-
2	**1a** (I, Boc)	Co(acac)_3_	TMEDA	**1a**/**2a** = 21:79	n. d.
3	**1a** (I, Boc)	Co(acac)_3_	TMCD	**1a**/**2a** = 13:87	n. d.
4	**1a** (I, Boc)	CoCl_2_	TMCD	**1a**/**2a** = 0:100	**2a** (81%)
5^[d]^	**1b** (I, Ts)	CoCl_2_	TMCD	**1b**/**2b** = 0:100	**2b** (69%)
6 ^[d], [e]^	**1c** (I, Bn)	CoCl_2_	TMCD	**1c**/**2c** = 0:100	**2c** (66%)
7 ^[d]^	**1d** (Br, Boc)	CoCl_2_	TMCD	**1d**/**2a** = 0:100	**2a** (83%)

^[a]^ TMEDA = *N*,*N*-tetramethylethylenediamine, TMCD = (*R*,*R*)-tetramethylcyclohexanediamine. ^[b]^ The ratio was determined using the crude ^1^H-NMR spectrum. ^[c]^ Isolated yield. ^[d]^ Two equivalents of PhMgBr were added. ^[e]^ 50 mol % of TMCD were added.
